# Circummandibular Wiring of Symphysis Fracture in a Five-Year-Old Child

**DOI:** 10.1155/2013/930789

**Published:** 2013-06-24

**Authors:** Krishna Priya Vellore, Srinivas Gadipelly, Brahmananda Dutta, Vijay Bhaskar Reddy, Sri Ram, Arun Parsa

**Affiliations:** ^1^Department of Pedodontics and Preventive Dentistry, MNR Dental College and Hospital, Andhra Pradesh, Sangareddy 502001, India; ^2^Department of Oral and Maxillofacial Surgery, MNR Dental College and Hospital, Andhra Pradesh, Sangareddy 502001, India

## Abstract

The treatment of pediatric maxillofacial fractures is unique due to the psychological, physiological, developmental, and anatomical characteristics of children. *Method*. This case report describes the management of symphysis fracture of mandible in a 5-year-old boy. The fracture was treated by acrylic splint with circummandibular wiring. *Results*. The splint was removed after 3 weeks. The patient had no complaints, and radiograph revealed a healed fracture. *Conclusion*. The clinical outcome in the present case indicates the management of mandibular fractures in pediatric patients using acrylic splint with circummandibular wiring.

## 1. Introduction

Mandibular fractures are the most common (56%) facial skeletal injury in hospitalized pediatric trauma patients [1, 2]. Boys are affected twice as frequently as girls [2, 3]. Dentoalveolar injuries are more frequent facial injury (60%) in children (especially under the age of 5) but rarely require hospitalization. In pediatric patients symphysis and parasymphysis fractures account for 15%–20% and body fracture rare [4]. The treatment choice of fractures in the pediatric mandible depends on the age and the state of tooth development. 

Major injuries affecting the face are associated with hyperactivity of the child, fall, road traffic accidents (RTA), assault, and child abuses which are the most frequent risks of facial bone fractures in children [5]. 

Majority of the body and symphysis fractures in children are undisplaced because of elasticity of mandible and embedded tooth buds that holds the fragments together “like glue” [6, 7]. If displaced, closed reduction and immobilization are performed. 

The following paper will review the triage, evaluation, and management of facial trauma in children. It highlights the role of acrylic splint with the use of circummandibular wiring technique in the management of symphysis fracture in a 5-year-old child. 

## 2. Case Report 

A 5-year-old male child reported to the Outpatient Department of Pedodontics and Preventive Dentistry, MNR Dental College and Hospital, Sangareddy, India, with a history of fall from steps. The patient was conscious, not well oriented with dressings in the lower jaw andno history of convulsions, vomiting, and bleeding from ear and nose. Haematological parameters such as blood count, CT, and BT were normal at the time of examination.

## 3. Clinical and Radiological Examination

**Figure 1:**
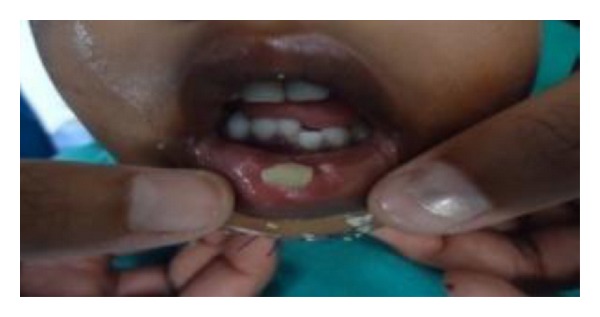
Preoperative photograph showing symphysis fracture.

Extraoral examination revealed the presence of a swelling in the anterior region of mandible. There was limited mouth opening because of pain and possible muscle spasm. On intraoral examination, bleeding was evident within the mouth. All primary teeth were present. A clinically minimally displaced fracture of the mandible in the area between two mandibular primary central incisors was noted. This resulted in an altered occlusion and a midline diastema in the fractured area. The examination also revealed a subluxation of the upper right primary incisor which was not treated as followup is required and a deep laceration on the chin (Figure 1). 

**Figure 2:**
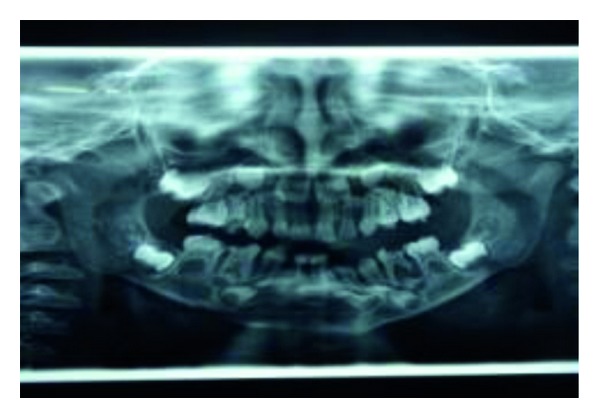
Preoperative OPG.

**Figure 3:**
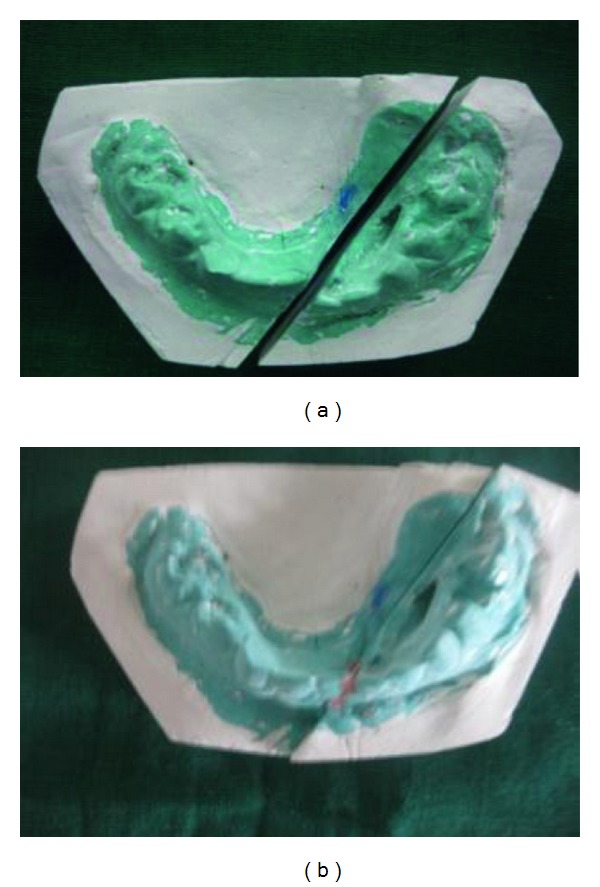
Preoperative model setup.

**Figure 4:**
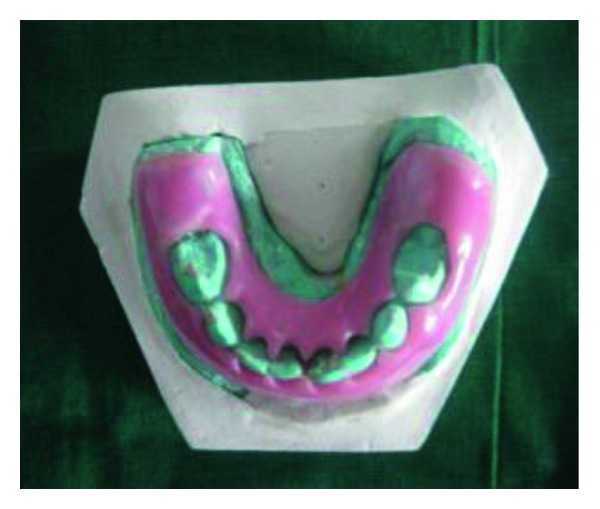
Prefabricated surgical splint on the model.

In the radiographs, mandibular symphysis fractures were evident between lower central incisors (Figures 2 and 9). Impressions of both jaws were taken with alginate impression material before mandibular reduction (Figure 3). An acrylic cap splint was then constructed on the model of the patient's arches after reducing the fracture on the models (Figure 4). 

## 4. Management

**Figure 5:**
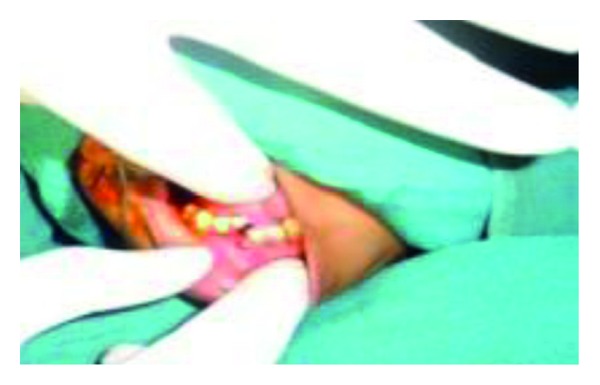
Intraoperative reduction of the segments.

**Figure 6:**
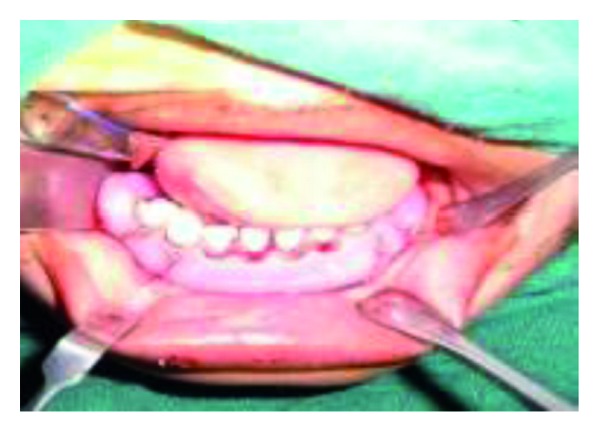
Intraoperative photograph showing splint in position, secured with circummandibular wires.

Under general anaesthesia, the dislocated segments were reduced by bidigital pressure with the guidance of the surgical splint (Figure 5). A small stab incision was placed at the inferior border of the mandible in the right side. A William velsey Fry awl was introduced through the stab incision. The bone awl was guided along the body of the mandible and taken out lingually. Next the wire was tied in and the awl was gently guided along the lower border of the mandible and passed into the buccal sulcus (Figure 6). 

The wire was held together and ironing was done to adapt the wire in close approximation to the bone. This also prevents soft tissue injury and an unesthetic scar. The acrylic cap splint was then stabilized on the right side by winding the wire in a clockwise direction. The same procedure was followed on the left side. Care was taken to avoid pulling the wire through the mandible since the child was young and at this stage the mandibular cortex is thin and relatively less dense. The external wound on the chin was thoroughly debrided and primary closure was done in two layers. 

**Figure 7:**
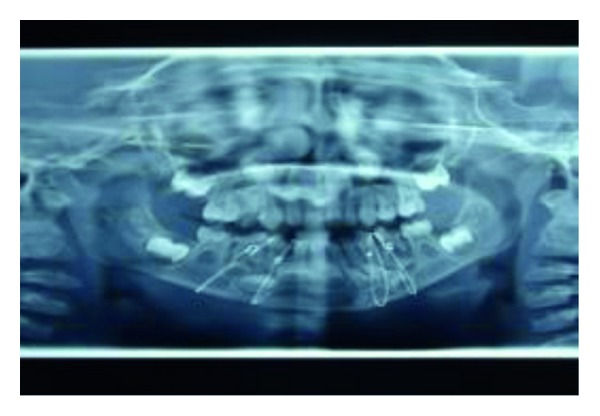
Intraoperative OPG with circummandibular wiring.

**Figure 8:**
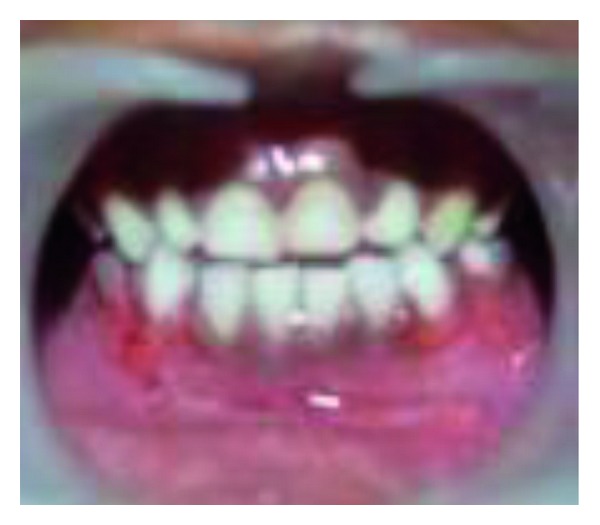
Postoperative followup.

**Figure 9:**
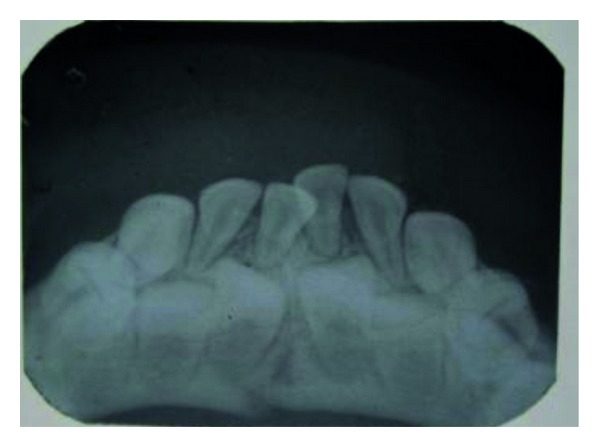
Preoperative IOPA of 73–83 region showing displaced 71 and 81.

**Figure 10:**
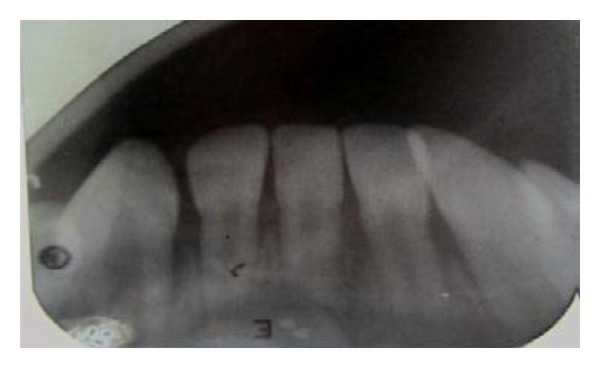
Postoperative IOPA of 73–83 region after 3 weeks with well-aligned 71 and 81.

OPG radiograph was taken postoperatively to check if the wires were properly secured to bone (Figure 7). Postoperative antibiotic treatment was started for 1 week. Soft diet, avoidance of physical activities, and antibacterial mouth rinse were prescribed. Postoperative monitoring was performed on a weekly basis and was favourable in both healing and function. No signs of complications were observed during the healing period (Figures 8 and 10). The interdental wiring and acrylic splint were removed after 3 weeks. 

## 5. Discussion

Fractures of the mandible comprise the most common facial skeletal injury among hospitalised pediatric trauma patients. They are the second most common facial skeletal injury, behind nasal fractures in the general pediatric population [8–11]. Boys outnumber girls in the incidence of mandible fractures by a ratio of two in one, with falls, blunt trauma, and motor vehicle accidents cited as the most common causes [10–12]. The areas of pediatric mandible that are most frequently fractured are in condyles, subcondylar, and angle regions (80%), and the symphysis/parasymphyseal area (15% to 20%) fractures of the body of the mandible are rare in pediatric population [10]. 

Factors to be considered in the definitive treatment of the dentoalveolar injury include (1) age and cooperation of the patient; (2) duration between trauma and treatment; (3) location or extent of the injury; (4) injury to primary or permanent dentition; (5) stages of root development; (6) presence of fracture of supporting bone; and (7) periodontal health of remaining teeth [13]. Among the commonly used treatment options, acrylic cap splints are ideal. They avail support not only from the adjacent teeth but also from bone. They are easy to fabricate and are economical. Routinely, they are used in stabilising mandibular fractures, as they can be stabilised by the use of circum-mandibular wire. 

## 6. Conclusion

“Facial fracture in children is a common type of injury suffered by pediatric patients. Causes and patterns of facial fractures vary with age. Knowledge of the association of dental injuries and maxillofacial fractures is a basic tool for their prevention. The majority of these fractures can be managed conservatively. The results of the fracture treatment presented here verified the usefulness of open cap splint in cases of mandibular body, parasymphysis, symphysis fracture.”
